# The Werner syndrome protein: linking the replication checkpoint response to genome stability

**DOI:** 10.18632/aging.100293

**Published:** 2011-03-07

**Authors:** Pietro Pichierri, Francesca Ammazzalorso, Margherita Bignami, Annapaola Franchitto

**Affiliations:** ^1^ Genome stability group; ^2^ Section of Experimental and Computational Carcinogenesis; ^3^ Section of Molecular Epidemiology, Department of Environment and Primary Prevention, Istituto Superiore di Sanitá, Rome, Italy

**Keywords:** Werner syndrome protein, replication fork arrest, replication checkpoint, genome instability

## Abstract

The Werner syndrome protein (WRN) is a member of the human RecQ family DNA helicases implicated in the maintenance of genome stability. Loss of WRN gives rise to the Werner syndrome, a genetic disease characterised by premature aging and cancer predisposition. WRN plays a crucial role in the response to replication stress and significantly contributes to the recovery of stalled replication forks, although how this function is regulated is not fully appreciated. There is a growing body of evidence that WRN accomplishes its task in close connection with the replication checkpoint. In eukaryotic cells, the replication checkpoint response, which involves both the ATR and ATM kinase activities, is deputed to the maintenance of fork integrity and re-establishment of fork progression. Our recent findings indicate that ATR and ATM modulate WRN function at defined steps of the response to replication fork arrest. This review focuses on the novel evidence of a functional relationship between WRN and the replication checkpoint and how this cross-talk might contribute to prevent genome instability, a common feature of senescent and cancer cells.

## INTRODUCTION

**Figure 1.**
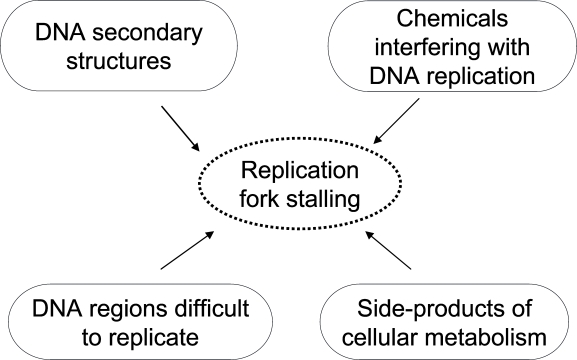
Summary of the potential sources of replication fork stalling

Maintenance of genome stability, an important issue in cancer biology and aging, relies on an accurate response to replication stress. During DNA replication several events can pose a serious threat to chromosomal integrity by interfering with fork stability. Indeed, a wide variety of sources can lead to fork stalling, a very frequent event occurring during S-phase (Figure 1). These include endogenous side-products of cellular metabolism, exogenous agents capable to interfere with DNA replication, as well as intrinsic structural features of specific genomic regions, such as the common fragile sites (CFS). Whenever arrested replication forks are not properly handled, cells may accumulate chromosomal rearrangements, as frequently observed in cancers and in a subset of genetic diseases characterized by chromosome fragility such as Werner, Bloom and Seckel syndromes, and Ataxia-telangiectasia [1]. To minimize this risk, eukaryotic cells have evolved a sophisticated apparatus deputed to the resolution of problems arising at replication forks: the replication checkpoint. This is an essential tool for safeguarding genome stability that brings together replication, repair and cell cycle proteins in a coordinated network having the ATR as the main controller. The observed defects of checkpoint functions in cancer cells highlight the importance of this mechanism of protection in mammalian cells as a barrier against uncontrolled cell proliferation [2-4]. Furthermore, several studies have revealed that the accumulation of DNA damage during life-time may be the major driving force of the aging process. In agreement with this hypothesis, the activation of the checkpoint response is observed in aging tissues [5].

### The replication checkpoint response

The link between replication defects, cancer and aging underscores the importance of an efficient and accurate monitoring of genome integrity during the S-phase. This is probably the reason why there are multiple checkpoint activities in the S-phase of the cell cycle [6, 7]. Moreover, the presence of multiple, and to some extent, redundant checkpoints, may be explained by the complexity of the DNA duplication process because of natural or accidental impediments. For instance, the human genome contains several regions, such as fragile sites, that show high propensity to form DNA secondary structures and are considered naturally-occurring replication fork stalling sites. Typically, the replication checkpoint determines both local actions at stalled forks as well as scattering of the signal to prevent premature entry into mitosis until DNA replication is completed, the so-called S/M checkpoint response [6-8]. Here, we will focus our attention on the local activity of the replication checkpoint, the one that stabilizes and protects replication forks from collapse and accumulation of potentially-harmful double strand breaks (DSBs) [9]. The key protein of the replication checkpoint is the ATR kinase that senses unusually-long stretches of RPA-coated single-stranded DNA (ssDNA), produced by the uncoupling of replicative helicase and transiently blocked DNA polymerases [10]. ATR forms a stable complex with ATRIP (ATR-interacting protein), which recruits ATR at stalled fork and is essential for ATR signaling. Activation of ATR-dependent pathway requires also the mediator protein TopBP1, which directly stimulates the kinase activity of the ATR-ATRIP complex towards several substrates. Recruitment of TopBP1 depends on the RAD9/RAD1/HUS1 (9.1.1) complex that is, in turn, loaded by the clamp loader RAD17/RFC2-5. The 9.1.1 complex interacts through the phosphorylated C-terminal tail of RAD9 with the BRCT domain of TopBP1 [11], leading to the activation of the ATR kinase [12]. Claspin, another checkpoint mediator protein, together with RAD17 facilitates ATR phosphorylation of CHK1 [13]. Once phosphorylated, CHK1, a critical effector of the replication checkpoint, is released from the chromatin and activates downstream substrates for promoting stalled fork stabilization, slowing-down of cell cycle progression and prevention of the S/M transition.

Several DNA repair proteins are also substrates of ATR, although it is unclear how these proteins contribute to the maintenance of fork integrity and, most importantly, which is the functional relevance of these ATR-dependent phosphorylation events.

The preferred target of ATR activity is a minimal consensus Ser/Thr-Gln (S/TQ) motif, often found clustered in checkpoint and DNA repair proteins such as CHK1, BRCA1, FANCD2, BLM and WRN. Interestingly, ATR shares many substrates with the related checkpoint kinase ATM, providing evidence of a cross-talk between ATR- and ATM-dependent pathways. The different phenotypes associated with loss of ATR or ATM demonstrate however, that these enzymes do not have redundant functions.

### The Werner syndrome protein

Werner syndrome protein (WRN), one of the five members of the human RecQ family of DNA helicases, shows helicase and exonuclease activities, both widely implicated in the maintenance of genome stability. Mutations in the WRN gene give rise to a severe human disease: the Werner syndrome (WS). WS is an inherited disorder in which affected individuals exhibit features of accelerated aging in early adulthood such as bilateral cataracts, greying of the hair, wrinkled skin, osteoporosis, type II diabetes, atherosclerosis, cardiovascular disease, as well as a high incidence of various neoplasms, including different types of carcinomas and sarcomas [14, 15]. Cells derived from WS patients display a reduced lifespan and an S-phase prolongation, in agreement with a fundamental role of this enzyme during DNA replication. Another hallmark of WS cells is an elevated genomic instability manifested as spontaneous chromosomal abnormalities and large deletions in many genes [16, 17], which may represent an important determinant of the increased risk of cancer and of the aging phenotype [18-20]. Previous studies demonstrated that lack of functional WRN results in high spontaneous yield of DNA breakage, an observation consistent with the observed high rate of chromosomal rearrangements [21]. Recent data indicate that loss of WRN leads to the activation of an alternative pathway of fork recovery resulting in DSBs accumulation that are next repaired through recombination [22]. These findings support the hypothesis that, upon replication fork stalling, WRN acts to limit fork collapse and/or to promote repair of DSBs.

*In vitro* studies demonstrate that the WRN helicase activity can unwind G4-tetraplex structures of the Fragile X syndrome repeat sequence d(CGG)n and other DNA secondary structures such as hairpins or forked DNA, more efficiently than double-stranded duplex DNA. WRN can also catalyse branch migration of Holliday junctions and melting of D-loops, which represent recombination intermediates. On the other hand, the WRN exonuclease activity acts preferentially on DNA structures such as bubble, loop, stem-loop and 3- or 4-way junction DNA. Based on these biochemical activities of WRN, it is thought that *in vivo* WRN participates in replication, recombination and repair or in a combination of these processes such as recombination during replication. Thus WRN might be implicated in the resolution of DNA secondary structures that can be formed during all the above-mentioned processes. In agreement with this hypothesis, WRN binds and/or functionally interacts with several proteins involved in DNA transactions. For instance, RPA physically interacts with WRN *in vitro*, stimulates its helicase activity, and, following HU exposure, co-localizes with WRN at replication fork stalling sites and assists WRN in the resolution of replication arrest. Co-immunoprecipitation experiments suggest that WRN and RPA association is enhanced in response to fork blockage inducing-treatments and this interaction is instrumental for the WRN-mediated displacement of RPA from DNA that contributes to fork recovery [23]. Moreover, it has been established that WRN participates in a multi-protein complex including ATR and the recombination proteins RAD51, RAD52, RAD54 and RAD54B, supporting a role for WRN in the later steps of the HR process [24].

The pleiotropic nature of WRN and the multiplicity of interactions make very difficult to determine the prominent biological function of this protein and to correlate loss of a specific activity, meant as biological and not only enzymatic, with the cellular and organismal premature senescence that characterizes the WS syndrome. However, since almost all the WS cellular phenotypes have a strong connection with defective DNA replicative processes, there is a wide consensus on a role of WRN as a replication caretaker, probably acting as an integral factor of the checkpoint response acting in the S-phase of the cycle.

### The cross-talk between WRN and the replication checkpoint

Several studies from our and other groups envisaged a possible cross-talk between WRN and ATR. In response to replication stress, WRN undergoes phosphorylation in an ATR/ATM-dependent manner and co-localizes with ATR at nuclear foci [25]. In addition to this, WRN has been found to interact or co-localize with proteins involved either in the intra-S or replication checkpoint, such as ATR or the MRE11 complex [25-28]. Of particular interest is that WRN helicase activity and ATR-mediated checkpoint response collaborate in a common pathway to maintain CFS stability [29]. These findings reinforce the hypothesis that WRN plays an essential role in the maintenance of genome stability by repairing damaged forks, whenever they stall, most likely in collaboration with ATR.

**Figure 2.**
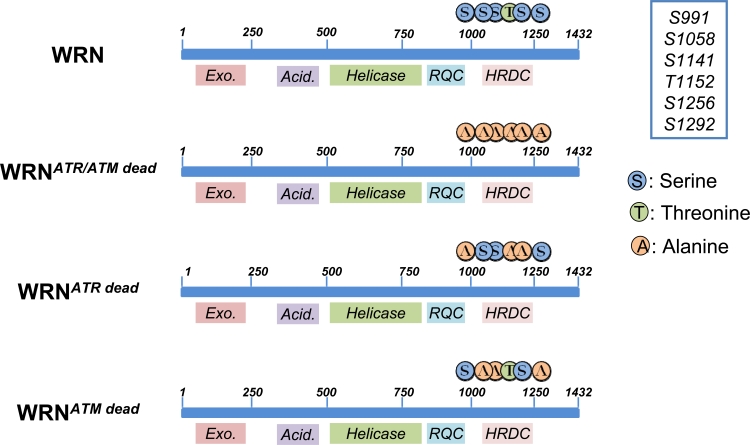
Schematic representation of ATR/ATM-phosphorylation sites clustered on the C-terminal region of wild-type (WRN) or mutant forms of WRN (WRN^ATR/ATMdead^, WRN^ATRdead^ and WRN^ATMdead^). Locations of Alanine substitutions are indicated

Although the detailed mechanism(s) is not fully appreciated, WRN might facilitate resumption of replication either by processing intermediates to avoid unscheduled recombination or alternatively by promoting recombination [30, 31]. However an open question is: how could WRN function be regulated? As reported for several DNA damage response proteins, post-translational modifications of WRN are good candidates for regulating its activity. The presence of ATR/ATM minimal consensus sequences at the C-terminus of WRN (Figure 2) are suggestive of a direct relationship between ATR/ATM and WRN. Although previous works included WRN among the putative ATR/ATM targets, no data on the functional consequences of WRN phosphorylation during the recovery of stalled forks have been reported.

In our recent study, the identity of the ATR/ATM phosphorylation sites were identified by site-directed mutagenesis of critical residues in the WRN protein [32]. These experiments also demonstrated that ATR and ATM do not target the same residues, suggesting functional differences between the various phosphorylation events. Thus biochemical studies evidenced that ATR phosphorylates the S991, T1152 and S1256 residues, while ATM specifically targets S1141, S1058 and S1292 [32]. We do not know if there is any hierarchy in the phosphorylation of the three residues by ATR, but expression of an N-terminal-truncated fragment of WRN in HEK293T cells evidenced that the C-terminal region of WRN exists as multiple phosphorylated isoforms, indicating that more than one residue, or a combination of residues, is phosphorylated *in vivo*. Noteworthy, two of the ATR-targeted residues (S991 and S1256) are conserved in WRN homologues from other species (e.g. in X. laevis FFA-1 or mouse WRN). This together with their location within domains involved in DNA-protein association or protein-protein interaction, is a strong clue for a crucial functional role [33]. These observations prompted us to analyze the role of those phosphorylation events during the response to perturbed replication. To this aim we generated cells stably expressing three different WRN phospho-mutants: WRN^ATRdead^, WRN^ATMdead^ and a double mutant WRN^ATR/ATMdead^, (Figure 2; [32]). One of the most striking feature of WRN is its ability to relocalize in nuclear foci at replication fork stalling sites. It has been long speculated on the mechanism(s) regulating WRN subnuclear dynamics [34] and in the last few years the role of post-translational modifications of this protein (mainly phosphorylation and acetylation) has become more evident. Indeed, both acetylation and c-Abl-mediated phosphorylation have been correlated to WRN delocalization from nucleoli into DNA damage-induced foci [35, 36], but the mechanism underlying building-up of WRN at fork stalling sites remained elusive. Our data indicate that accumulation of WRN at stalled replication foci is clearly regulated through ATR-dependent phosphorylation. Indeed, either the WRN^ATRdead^ or the WRN^ATR/ATMdead^ mutant shows a reduced ability to form nuclear foci after replication arrest, whereas loss of the ATM-dependent phosphorylation does not affect WRN re-localization. However, abrogation of ATR phosphorylation sites does not abolish completely WRN accumulation at stalled forks. This partial phenotype suggests that phosphorylation might be involved in stabilizing the binding of WRN to DNA or to an interactor mediating DNA binding. Consistently, our unpublished observations indicate that, after HU treatment, low salt concentrations are sufficient to release WRN^ATR/ATMdead^ from chromatin, supporting the hypothesis that ATR-dependent phosphorylation of WRN may stabilize WRN binding to the chromatin once it has been recruited. Interestingly, upon replication arrest, WRN re-localization is completely abrogated in cells depleted of the 9.1.1 complex (Pichierri *et al*., submitted), suggesting that the replication checkpoint controls WRN function at stalled forks acting at multiple levels. Further supporting the possibility that ATR-dependent phosphorylation may be required to fasten WRN at stalled forks, phosphorylation and ability to form nuclear foci are two separable events. Indeed, while 9.1.1 complex down-regulation prevents both assembly of WRN nuclear foci and phosphorylation, depletion of TopBP1 reduces WRN phosphorylation without affecting its localization in nuclear foci (Pichierri *et al*., submitted). Altogether, it seems likely that phosphorylation of WRN follows its recruitment at sites of stalled forks, probably to “activate” fork processing. It is tempting to speculate that phosphorylation of WRN might affect separately helicase or exonuclease activity.

It is well recognized that, in response to replication stress, the main function of the replication checkpoint is to maintain fork integrity, promoting fork recovery and then replication restart. Indeed, loss of ATR leads to accumulation of DSBs and chromosome breakage [37-41], all features also observed in WRN-deficient cells [21, 22].

Using cells expressing the three WRN phospho-mutants, we find that ATR-dependent phosphorylation is functionally-related to the ability of WRN to prevent DSBs accumulation upon replication arrest. Thus, early after replication arrest, loss of ATR, WRN, or ATR phosphorylation sites in WRN determines degeneration of stalled replication forks into DSBs. Furthermore, down-regulation of ATR in cells expressing the WRN^ATR/ATMdead^ mutant, highlights a more intimate cross-talk between WRN and ATR. Indeed, ATR RNAi does not further enhance DSBs accumulation in cells expressing the ATR-unphosphorylable form of WRN, indicating that the well-described ATR-dependent stabilization of stalled forks is basically carried out through phosphorylation and regulation of WRN by ATR. Checkpoint activation and cell cycle arrest, however, are not affected by abrogation of ATR phospho-sites in WRN (WRN^ATRdead^), but requires ATR depletion. This is in agreement with the multiple activities regulated by ATR and with the more severe phenotype of ATR-deficient cells [28, 32]. Interestingly, loss of ATR-dependent phosphorylation of WRN also affects stability of the human natural hot-spots of replication arrest, the CFS ([29]; Franchitto and Pichierri, unpublished).

While ATR-dependent phosphorylation is functionally-related with WRN protective role against DSB formation at stalled forks, phosphorylation of WRN by ATM appears to be involved in some additional aspects of the response to replication arrest. Indeed, we observed that mutation of both ATR and ATM phosphorylation sites in WRN (WRN^ATR/ATMdead^) determines a higher accumulation of DSBs and a reduced restart of stalled forks compared to mutation of the ATR phosphorylation sites. Moreover, cells expressing the WRN^ATR/ATMdead^ die after attempting recovery from replication arrest. Thus if ATR regulates the prevention by WRN of fork breakage, is it possible that ATM modulates a role of WRN during repair of DSBs formed at collapsed forks? The extensive cell death observed in WRN^ATR/ATMdead^ cells, which is greater than that of cells lacking WRN [28] or expressing the ATR-unphosphorylable WRN allele (WRN^ATRdead^), is consistent with this hypothesis. It is well known that RAD51-dependent recombination is the principal pathway involved in the repair of replication-associated DSBs [42] and that WS cells show up-regulation of RAD51 foci and enhanced engagement of recombination after replication arrest [21, 22]. Although WRN^ATR/ATMdead^ cells are more sensitive than WS cells to HU-induced replication arrest and exhibit a similarly high percentage of DNA breakage accumulation, levels of RAD51 foci are comparable to those observed in wild-type cells [32]. Moreover, although RAD51 depletion affects viability of WS cells after prolonged replication arrest (Franchitto *et al*., unpublished), down-regulation of RAD51 in WRN^ATR/ATMdead^ cells does not result in a comparable reduction of viability. This suggests that the WRN^ATR/ATMdead^ mutant acts in a dominant-negative way interfering with the activation of both the WRN- and RAD51-dependent pathways involved in fork recovery. This finding is intriguing because the WRN^ATR/ATMdead^ allele should require up-regulation of RAD51 foci forming activity vis-à-vis to the extensive DSB formation at blocked forks. Thus it is possible that ATM-dependent WRN phosphorylation might influence the ability of RAD51 to form foci by interfering with HR-mediated replication restart. How the ATM-dependent phosphorylation of WRN may interfere with RAD51 foci formation is again a matter of subnuclear dynamics. Indeed, the WRN^ATR/ATMdead^ protein has a quite schizophrenic subnuclear dynamics: it shows reduced accumulation at nuclear foci shortly after replication arrest, but it is found localized in foci more than in its wild-type counterpart after prolonged replication arrest or after recovery from that arrest. Such a biphasic behaviour is clearly dependent on loss of ATM phosphorylation, since the WRN^ATRdead^ protein does not show the persistence in nuclear foci observed for the unphosphorylable protein (WRN^ATR/ATMdead^). Interestingly, the late- WRN^ATR/ATMdead^ foci co-localize extensively with the DSBs marker γ-H2AX. Collectively, these findings tell us that the persistence of WRN at collapsed forks counteracts accumulation of RAD51 foci. At this stage we do not know if RAD51 is prevented from accumulating at collapsed forks simply because WRN hinders the DNA ends or rather because WRN persistence at DSBs dismantles actively the RAD51 nucleofilaments. A recent report from Patrick Sung's lab indicates however that WRN, differently from other RecQ helicases, fails to disrupt RAD51 nucleofilament [43]. Further investigations are required to define how ATM-dependent phosphorylation of WRN favours RAD51 activity at collapsed forks.

### Concluding remarks and perspective

**Figure 3.**
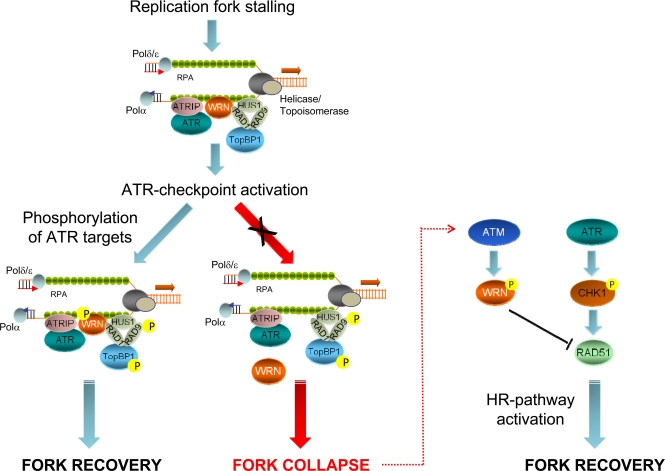
Role of ATR- or ATM-dependent modulation of WRN function in promoting correct recovery from replication arrest In response to replication fork stalling, the ATR-ATRIP and 9.1.1 complexes are independently loaded at RPA-bound ssDNA regions to activate the replication checkpoint. Early after checkpoint activation, WRN is recruited to fork stalling sites through its interaction with RAD1, a subunit of the 9.1.1 complex. This stage corresponds to formation of WRN foci, which co-localise with RPA. After 9.1.1-dependent relocalisation in foci, WRN is phosphorylated by ATR in a manner that could be dependent on TopBP1. Phosphorylation of WRN by ATR is instrumental for preventing DSBs accumulation at stalled forks and and ensuring faithful recovery of replication forks. Degeneration of the stalled forks into breakage, such as in the absence of ATR phosphorylation of WRN, can cause the activation of an alternative pathway: in this case, ATM-dependent phosphorylation promotes de-localization of WRN from collapsed forks to prepare the way for RAD51-mediated replication recovery, which is also dependent on RAD51 phosphorylation by CHK1.

Previous studies demonstrated that both ATR and ATM are necessary for recovery from replication-dependent DSBs formation and to regulate RAD51 foci assembly [44-46]. Our results show that the primary function of ATR in the prevention of DSBs accumulation at stalled forks is carried out by WRN, while recovery from fork collapse and DSBs generated at blocked forks is strongly affected by loss of ATM-dependent WRN phosphorylation. Thus, our findings suggest that a timely and accurate regulation of the WRN protein by ATR and ATM is crucial to maintain viability and genome stability in human cells under perturbed replication (Figure 3). Interestingly, engagement of RAD51-dependent recombination at collapsed forks seems the most finely-tuned step in which both ATR and ATM collaborate. Indeed, after replication stress, RAD51 relocalization in foci is regulated by ATR through CHK1-dependent phosphorylation [44] and, consistently, depletion of ATR by RNAi results in a low percentage of RAD51 foci in wild-type or WS cells [32]. However, in the absence of the ATM-dependent WRN phosphorylation efficient RAD51 phosphorylation does not prevent cell death. Thus, ATM-dependent WRN phosphorylation somehow functions as “licensing” event in RAD51-dependent fork recovery. Such a complicate regulation of the stability of stalled forks and of the replication fork reaction is not unexpected and tight control of recombination is likely correlated with the harmful effect of unscheduled recombination on genome stability. A similar and explicative situation has been described in fission yeast, where the association of the recombination protein MUS81 with stalled forks is actively prevented by Cds1-dependent phosphorylation, and unphosphorylable mutants show persistent chromatin localization [47].

WRN is a pleiotropic protein with lots of interactors [33] and our WRN phosphorylation mutants seem to have the ability to interfere specifically with single biological function of WRN, thus probably affecting well defined protein-protein interactions. Thus, these new WRN alleles could allow a detailed analysis of the cross-talk between the replication checkpoint and WRN. Moreover, they could be a useful tool to determine the identity of the molecular mechanisms underlying premature replicative senescence of WS cells and, by extension, through the use of mouse models, they could answer the question if the premature aging phenotype of WS derives from abnormal accumulation of DNA damage.
